# Vaccinations for older adults

**DOI:** 10.2471/BLT.22.288550

**Published:** 2022-06-01

**Authors:** Jane Barratt

**Affiliations:** a International Federation on Ageing, 1 Bridgepoint Drive, G.238 Toronto, M4M2B5 Ontario, Canada.

Global population ageing affects the social, health and economic fabric of every country. Older adults (60 years and older) are at risk of vaccine-preventable diseases including influenza, pneumococcal pneumonia, shingles and pertussis.^1^ The coronavirus disease 2019 (COVID-19) pandemic resulted in the interruption and de-prioritization of routine immunization schedules. The pandemic has also exposed the fragile nature of health systems, the low levels of investment in prevention strategies including immunization, and the failure to expand immunization for older people through collaborations with other sectors, namely civil society and business ‒ inside and outside the health system.

Vaccination is a major driver in advancing life expectancy because it reduces deaths from complications of coexisting chronic conditions and directly from infectious diseases.^2^ National immunization plans largely focus on reducing mortality and morbidity in children, although the major burden of vaccine-preventable diseases is in adults.^3^

Fostering healthy ageing is a hallmark of the United Nations (UN) Decade of Healthy Ageing 2021–2030, yet the coverage of adult vaccination is suboptimal worldwide.^4^ In this issue of the *Bulletin*, Rizvi and Singh used data from the first Longitudinal Ageing Study in India, and found that vaccination coverage among older Indian adults (45 years or older) for influenza, pneumococcus, typhoid and hepatitis B was 2% or less and varied by socioeconomic, demographic, health and residence-related characteristics.^5^

Global policies on adult immunization are neither sufficient nor adequate. A study to assess adult immunization programmes in Member States of the World Health Organization (WHO) found that 38.7% (2.93 billion) of the world’s population live in a country reporting at least one adult immunization programme. Influenza vaccine programmes were reported by 58.8% (114/196) of countries, while other vaccines such as pneumococcal, herpes zoster and hepatitis B were much less common.^6^

Why countries are consistently below national adult immunization targets, why no national target exists or why the target only applies to influenza vaccination are critical policy questions to answer, as low coverage of relevant vaccines persists in the older population.

Various studies have identified modifiable barriers to vaccination in older adults, such as the lack of public awareness on the benefits of vaccines, misconceptions of adult vaccination and healthy ageing, limited knowledge of funded vaccines and logistical issues related to vaccine delivery, including insufficient supply of age-specific vaccines, complex vaccination procedures, the inability to determine timing and type of vaccination and lack of funding for vaccines or vaccine visits.^7^^,^^8^

The COVID-19 pandemic has underscored the critical nature of integrated policy development across sectors, disciplines and within intergovernmental agencies. The WHO Immunization Agenda 2030, the UN Decade of Healthy Ageing,^9^ the WHO *Global report on ageism*^10^ and *Defeating Meningitis by 2030: A Global Road Map*^11^ represent public health policy agendas with the potential to improve the health of people across the life course.

The magnitude and global scale of disruption to routine immunization and lack of concerted efforts to expand immunization at older ages evokes the potential dangers of vaccine-preventable disease outbreaks.^12^ The global health community needs to vigorously protect the progress made in immunization and ensure sustained investment across generations with attention to older adults.
